# Underweight and early childhood caries among young children in rural Cambodia: a pilot study

**DOI:** 10.1038/s41405-021-00089-y

**Published:** 2021-09-08

**Authors:** Yu Kubota, Nhep San Pech, Callum Durward, Hiroshi Ogawa

**Affiliations:** 1 Division of Preventive Dentistry, Faculty of Dentistry & Graduate School of Medical and Dental Sciences, Niigata University, Niigata, Japan; 2 Faculty of Dental Nurse, University of Kampong Cham, Kampong Cham, Cambodia; 3 Faculty of Dentistry, University of Puthisastra, Phnom Penh, Cambodia

**Keywords:** Paediatric dentistry, Dental epidemiology

## Abstract

**Objectives:**

To investigate the association between underweight and early childhood caries (ECC) among children aged one to three years in rural Cambodia.

**Materials and methods:**

A total of 200 Cambodian children aged one to three years at several villages in Kampong Cham province participated in this study. The children whose *Z* scores were below two and three standard deviations were considered as moderately-underweight and severely-underweight. Children whose mid-upper arm circumstance (MUAC) was below 12.5 cm, were regarded as having malnutrition. ECC was recorded following the WHO guidelines. Associated factors were obtained through interviews with caregivers.

**Results:**

33.5% and 7.0% of the children were identified as being moderately-underweight and severely-underweight, respectively. The prevalence of ECC was 61.5%. ECC was significantly associated with children being moderately-underweight (*P* < 0.05). The prevalence of ECC was higher prevalence of those who with severely-underweight, although significant differences were not observed (*P* = 0.054). Logistic regressions showed that those with low birth weight (OR = 2.57; 95% CI = 1.03–6.40) and malnutrition (OR = 4.71; 95% CI = 1.08–20.62) were likely to be moderately-underweight and severely-underweight, whereas those who with ECC had more moderately-underweight, although it was not significant (OR = 2.21; 95% CI = 0.97–5.00). Those with low birth weight (OR = 10.68; 95% CI = 2.95–38.65) and ECC (OR = 6.67; 95% CI = 1.02–43.61) were likely to be severely-underweight.

**Conclusion:**

The findings of this study suggest that low birth weight, malnutrition and ECC were associated factors of underweight in this population.

## Introduction

Children’s physical growth is rapid during the first three year after birth [1]. International recommendations are to breast feed exclusively for the first six months, after which complementary foods can be introduced into the diet, in a process called weaning. This coincides with the period of time when primary teeth start to erupt, normally starting with the lower incisors at about six months of age. Weaning continues until breast-feeding or bottle-feeding stops, usually before the age of three [2]. During this period, oral functions, including lip, tongue and jaw movements develop, and the eruption of the twenty primary teeth is complete [3]. This points to a possibly important interrelationship between nutrition, the growth and development of the child, and oral health.

Underweight (low weight-for-age) is a main indicator for malnutrition in children [4] and more common in children from rural areas possibly because of the influence of socioeconomic status (SES) and different dietary practices [5]. Cambodia shows a higher prevalence of underweight children than many other countries [6]. Although the economic situation is steadily improving in Cambodia, the main cause of death among children aged under five is malnutrition [7]. According to the recent Cambodia Demographic and Health Survey, 24% of children under the age of five classified as underweight, and five percent are severely underweight. The proportion of children who are underweight peaks during the weaning period [8].

Early childhood caries (ECC) is defined as the presence of one or more decayed (non-cavitated or cavitated lesions), missing (due to caries), or filled tooth surfaces in any primary tooth in a child 71 months of age or younger [9]. A previous study in Kampong Cham province showed that 70% of children had developed ECC by two years old, even before all the primary teeth had erupted [10]. Turton et al. found that the ECC prevalence was 85% in children aged three years who had complete primary dentitions [11]. By the time Cambodian children reach school, the average six-year-old has a mean dmft (decayed, missing and filled primary teeth), nine teeth, with almost no children having evidence of any dental treatments [12]. Poor oral health in young Cambodian children has been linked to a variety of associated factors [10, 13]. Previous studies have suggested that an integrated approach between general health and oral health will be essential if these oral health issues in young children are to be addressed in the future [14, 15].

The associations between body weight and oral health status of children have been reported in several studies in Asian countries. Several of these showed that children with severe dental caries were likely to be underweight [16, 17]. In Cambodia, several studies to explore associated factors for underweight in children have been conducted [18, 19], however, two of these studies targeted school children with complete primary or mixed dentitions. The aim of this study was to investigate the associations between underweight and oral health related factors among children aged one to three years of age in a rural area of Cambodia.

## Methods

### Study site and participants

This cross-sectional study was conducted in Steung Trang district, Kampong Cham province from May 2018 to January 2019, Cambodia. Initially, we recruited children aged one to three who had registered births with several villages whose families attending the Khpob Ta Nguon Health Center in Khpob Ta Nguon. Before commencing the study, we obtained the permission from the local health administrative district in Cambodia and the chiefs in each village. After explaining the purpose and the protocol of the study, we obtained written informed consent from the caregivers. Children who declined participation in the study and those who had less than eight erupted teeth were excluded. Data from 200 children (males = 111, females = 89; mean age = 27.00 ± 9.41 months; median = 26.50 months) were analyzed. The sample size was calculated with the following formula.

*N* = Z^2^_α/2_ P(1 − P)/d^2^

*N* = required minimum sample size

Z_α/2_ = confidence level at 95% (1.96)

*P* = estimated prevalence of an indicator (proportion of underweight among 3-year-old children in Cambodia at 25%) [8]

d = maximum allowable error at 6%

*N* = (1.96)^2^(0.25)(1 − 0.25)/(0.06)^2^

 = 200.08

≒ 200

### Measurements of body weight and nutrition status

All parts of the survey were conducted in the Khpob Ta Nguon Health Center. Exact ages and birth weights of the children were obtained by referring to the birth registration records and their maternal health handbooks, respectively. Low birth weight was categorized as below 2500 g [20]. The current body weights of the children were measured with a digital weighting scale (TANITA BC-760, TANITA Inc, Tokyo, Japan) in units of 0.1 kg. These were plotted into the *Z* score curves (weight-for-age) following the WHO Child Growth Standards [21]. In the *Z* scores, below two (−2SD) and below three standard deviations (−3SD) were considered as moderately-underweight and severely-underweight, respectively [22]. To assess nutritional status, the mid-upper arm circumstance (MUAC) of each child was measured by using a non-stretch typed tape that UNICEF produced. MUAC is a simple and easy way to identify malnutrition and is widely adapted in the community level, and <12.5 cm was regarded as an indicator of malnutrition [23].

### Measurement of ECC

Before the oral examinations commenced, intra-examiner calibration was carried out on 20 subjects by one trained dentist. An obtained kappa score of 0.87 indicated a high level of agreement. The oral examinations were conducted under natural light with the examiner and mother sitting “knee-to-knee” and the child in the supine position on their laps. A visual examination of the teeth was conducted following the WHO Oral Health Surveys Basic Methods [24].

### Questionaire interview

The questionnaire interviews were conducted by local trained interviewers and included questions on socio-demographic background, and child-rearing practices, including dietary and oral hygiene practices related to ECC.

### Statistical analysis

Data was entered into an Excel spread sheet and transferred for statistical analysis using the statistical software package (SPSS, version 25.0, IBM Inc., Tokyo, Japan). The Shapiro–Wilk Test was performed to verify normal distribution of the data. The Chi-square test was used to investigate associations between variables and weight status. Logistic regression analyses were conducted by inputting variables with <0.1 at the baseline and adjusting for sex, age and the number of erupted primary teeth to determine the predictors of underweight among children. A *p* *<* 0.05 was set as indicating statistical significance.

### Ethical considerations

This study was conducted according to the guidelines of the Declaration in Helsinki and was approval by the Ethical Committee of Niigata University (2017-0187).

## Results

Table 1 shows the associations between body weight status and related factors. Among the 200 children in the study, 33.5% and 7.0% of them were identified as being moderately-underweight and severely-underweight, respectively. The prevalence of moderately-underweight children was 43.1% at the age of three and had significantly increased with age (*P* < 0.05). No significant differences were found between males and females. In this study, the prevalence of children with low birth weight was 15.2%. These children were significantly more likely to be moderately- (*P* < 0.05) and severely-underweight (*P* < 0.01) compared with the other children. 5.1% of children whose mid upper arm circumstance was <12.5 cm showed malnutrition. These children were significantly more likely to be moderately- and severely-underweight (*P* < 0.05).

**Table 1:** Association between underweight status and its related factors among young children (*n* = 200).

	Total (%)	Normal-weight (%)	Moderately-underweight (%)	*p*- value	Severely-underweight (%)	*p*- value
Variables	200 (100)	133 (66.5)	67 (33.5)		14 (7.0)	
Sex						
Male	111 (55.5)	78 (70.3)	33 (29.7)	0.21	8 (7.2)	0.90
Female	89 (44.5)	55 (61.7)	34 (17.0)		6 (6.7)	
Age						
1-year-old	78 (39.0)	61 (78.2)	17 (21.8)	0.018	5 (6.4)	0.30^d^
2-year-old	71 (35.5)	43 (60.6)	28 (39.4)		3 (4.2)	
3-year-old	51 (25.5)	29 (56.9)	22 (43.1)		6 (11.8)	
Household size^a^						
1–3	36 (18.1)	26 (69.4)	11 (30.6)	0.62	4 (11.1)	0.57^d^
4–6	137 (68.8)	88 (64.2)	49 (35.8)		9 (6.6)	
7–	26 (13.1)	19 (73.1)	7 (26.9)		1 (3.8)	
Main caregiver						
Mother	160 (80.0)	106 (66.3)	54 (33.8)	0.88	12 (7.5)	0.44^d^
Others	40 (20.0)	27 (67.5)	13 (32.5)		2 (5.0)	
Birth weight^b^						
<2.5 kg	30 (15.2)	14 (46.7)	16 (53.3)	0.01	8 (26.7)	<0.001^d^
≥2.5 kg	167 (84.8)	118 (70.7)	49 (29.3)		6 (3.6)	
Mid-upper arm circumstance^c^						
≦12.5 cm	10 (5.1)	3 (30.0)	7 (70.0)	0.017	3 (30.0)	0.025^d^
>12.5 cm	188 (94.9)	129 (68.6)	59 (31.4)		11 (5.9)	
Family income						
Under 100 $	100 (50.0)	67 (67.0)	33 (33.0)	0.99	8 (8.0)	0.41
100–250 $	79 (39.5)	52 (65.8)	27 (34.2)		6 (7.6)	
Over 250 $	21 (10.5)	14 (66.7)	7 (33.3)		0 (0)	
Birth order						
First	66 (33.0)	50 (75.8)	16 (24.2)	0.076	5 (7.6)	0.83^d^
Second	60 (30.0)	34 (56.7)	26 (43.3)		3 (5.0)	
Third and after	74 (37.0)	48 (66.2)	25 (33.8)		6 (8.1)	
Early childhood caries						
Yes	123 (61.5)	73 (61.2)	49 (39.8)	0.016	12 (9.8)	0.054
No	77 (38.5)	59 (76.6)	18 (23.4)		2 (2.6)	
Breast-feeding						
≦18 months	68 (34.0)	45 (66.2)	23 (33.8)	0.95	5 (7.4)	0.55^d^
>18 months	132 (66.0)	88 (66.7)	44 (33.3)		9 (6.8)	
Night breast-feeding						
≦18 months	77 (38.5)	52 (67.5)	25 (32.5)	0.81	6 (7.8)	0.73
>18 months	123 (61.5)	81 (65.9)	42 (34.1)		8 (6.5)	
Bottle-feeding						
≦18 months	98 (49.0)	66 (67.3)	32 (32.7)	0.80	4 (4.1)	0.11
>18 months	102 (51.0)	67 (65.7)	35 (34.3)		10 (9.8)	
Tooth-brushing						
≦18 months	50 (25.0)	29 (68.0)	21 (42.0)	0.14	3 (6.0)	0.52^d^
>18 months	150 (75.0)	104 (69.3)	46 (30.7)		11 (7.3)	
Sugary meal						
More than once a week	121 (60.5)	76 (62.8)	45 (37.2)	0.14	9 (7.4)	1.00^d^
Once a week	43 (21.5)	34 (79.1)	9 (20.9)		3 (7.0)	
Never	36 (18.0)	23 (63.9)	13 (36.1)		2 (5.6)	
Sugary beverage						
More than once a week	114 (57.0)	67 (58.8)	47 (41.2)	0.028	8 (7.0)	0.86^d^
Once a week	32 (16.0)	24 (75.0)	8 (25.0)		3 (9.4)	
Never	54 (27.0)	42 (77.8)	12 (22.2)		3 (5.6)	

^a^One missing data.

^b^Three missing data.

^c^Two missing data.

^d^Fisher’s exact test.

In this study population, 61.5% of children had ECC and all teeth with ECC were untreated. The children with ECC had a higher chance of being moderately-underweight than those children without ECC by 16.4% (39.8–23.4) (*P* < 0.05). Among child-rearing practices, no significant differences were observed between underweight status, and daytime and night-time breast-feeding after 18 months, bottle-feeding after 18 months, the introduction of tooth-brushing after 18 months and sugary food intake. However, more than once a weekly sugary beverage intake by children was significantly associated with moderately-underweight (*P* < 0.05). The children with high frequency of sugary beverage consumption had higher moderately-underweight status than those who did not by 20% (41.2–22.2).

Tables 2 and 3 show logistic analyses on underweight among children after adjusting for sex and age, and the number of erupted primary teeth. The children whose birth weight was low and who had a mid-upper arm circumstance of <12.5 cm were significantly more moderately-underweight (OR = 2.57; 95% CI = 1.03–6.40; *P* = 0.043, OR = 4.71; 95% CI = 1.08–20.62; *P* = 0.04), whereas those with ECC were more likely to be moderately-underweight, although this was not significant since the confidence interval included 1 (OR = 2.21; 95% CI = 0.97–5.00; *P* = 0.058) (Table 2).

**Table 2:** Logistic regression analysis on moderately -underweight among young children (*n* = 200).

Dependent variable				
Moderately -underweight				
Independent variables	S.E.	*p* value	Odds	95% CI
Sex				
Males (Ref)				
Females	0.36	0.22	1.56	0.77–3.14
Age				
1-year-old (Ref)				
2-year-old	0.61	0.13	2.50	0.76–8.24
3-year-old	0.73	0.16	2.79	0.67–11.54
Birth weight				
≥2.5 kg (Ref)				
<2.5 kg	0.47	0.043	2.57	1.03–6.40
Mid-upper arm circumstance				
>12.5 cm (Ref)				
≦12.5 cm	0.75	0.04	4.71	1.08–20.62
Birth order				
First (Ref)				
Second	0.45	0.06	2.35	0.97–5.73
Third and after	0.43	0.35	1.50	0.65–3.48
Early childhood caries				
No (Ref)				
Yes	0.42	0.058	2.21	0.97–5.00
Sugary beverage				
Never (Ref)				
Once a week	0.57	0.77	1.18	0.39–3.60
More than once a week	0.44	0.082	2.15	0.91–5.01

*S.E.* standard error, *CI* confidence interval.

**Table 3:** Logistic regression analysis on severely -underweight among young children (*n* = 200).

Dependent variable Severely -underweight				
Independent variables	S.E.	*p* value	Odds	95% CI
Sex				
Males (Ref)				
Females	0.65	0.72	1.26	0.36–4.45
Age				
1-year-old (Ref)				
2-year-old	1.13	0.61	0.56	0.06–5.10
3-year-old	1.30	0.76	1.50	0.12–19.03
Birth weight				
≥2.5 kg (Ref)				
<2.5 kg	0.66	<0.001	10.68	2.95–38.65
Mid-upper arm circumstance				
>12.5 cm (Ref)				
≦12.5 cm	0.93	0.071	5.41	0.87–33.76
Early childhood caries				
No (Ref)				
Yes	0.96	0.048	6.67	1.02–43.61

*S.E.* standard error, *CI* confidence interval.

Those with low birth weight and who had ECC were significantly more likely to be severely-underweight (OR = 10.68; 95% CI = 2.95–38.65; *P* < 0.001, OR = 6.67; 95% CI = 1.02–43.61; *P* = 0.048) (Table 3).

## Discussion

In the present study, we investigated the association between underweight status and ECC among children aged one to three years in a rural area of Cambodia. The prevalence of children who were moderately- and severely-underweight increased by three and two times, respectively, between the ages of one and three years. Previous cross-sectional surveys conducted in developing countries showed similar prevalence of underweight children which increased during the weaning period [5, 25]. According to a birth-cohort study in Nepal, the mean *Z* scores of weight-for-age steadily declined from birth to 8.5 years [26]. This suggests that early interventions from birth are essential to reduce the prevalence of underweight children.

In the present study, 15.2% of children were born with low birth weight and this was a clear predictor of underweight status. In the study population, this figure is higher compared with a previous study in rural Cambodia [27]. It is well-known that low birth weight is negatively associated with a variety of health outcomes, such as lower immune levels, a higher risk of diarrhea and undernutrition, and difficulties of catch-up in child body growth [28]. The latest national demographic and health survey in Cambodia revealed that the prevalence of low birth weight in rural areas was significantly higher than in urban areas, and that mothers with low education were more likely to give birth to low birth weight children [29]. The low literacy rate among rural Cambodian women is still a serious problem and an indicator of social and medical disadvantage; however, in recent decades, the literacy rate has been improving [30]. In rural Cambodia, female children are more likely to be deprived for opportunities of education than males, partly because they have to drop out of school to help their families with tasks, such as rearing their siblings and working in agriculture [31]. This limits their opportunities to learn about family planning, pregnancy and delivery, and child-rearing. In Cambodia, education of girls and young women is closely linked to social policies and environmental factors outside the control of individuals. Poor households lack resources to pay for schooling and associated costs, and to pay for clean water sources [32]. Previous studies have shown that mothers in the lower wealth quintile tended to deliver children with stunting [33] and a lower household index was a barrier to obtain maternal cares during pregnancy [34]. This suggests that education of girls and young women should be a priority to address the problem of low birth weight babies and underweight children in addition to addressing social determinants of health among rural children in Cambodia.

In this population, 5% of children had a MUAC below 12.5 cm and MUAC was an indicator of underweight. This finding was similar to that of the latest national demographic and health survey in Cambodia [35]. In Cambodia, deficiency of micronutrients, in particular, zinc, vitamin D and iodine, has been identified as a challenging issue among children [36]. Children with low MUAC have a higher risk of death [37] and should be a priority target group for interventions to improve nutrition and for nutritional education among pregnant women and mothers.

In a previous study on the same group of children, most children continued to breast feed (including at night) after 18 months [38]. WHO recommends exclusive breast-feeding for the first six months and continued breastfeeding for two years to enhance immunity and supply important nutrients for the growing infant [39]. However, a recent study on breast-feeding suggested that it can be difficult to provide all the necessary nutrients for a growing child by breast milk alone [40]. In Cambodia, it is estimated that 65% of mothers breasted for at least six months [8]. Many continue to breast feed well beyond this. There is also a high proportion of babies and infants who bottle-feed alongside or in place of breastfeeding. Prolonged nocturnal bottle- and breast-feeding has been associated with ECC, particularly if oral hygiene measures are not adequate [41].

In the present study, children with a frequent sugary drink intake had a higher prevalence of underweight. Sugary drinks are cheaper to buy than cows’ milk and infant formula, and are particularly harmful to the teeth. To foster child growth and development, healthy weaning practices are essential. These weaning practices can vary widely from country, largely influenced by sociocultural factors. In Cambodia, age-appropriate weaning which meets WHO recommendations should be promoted, taking into account the existing dietary culture and child-rearing environments.

Previous studies have shown that the oral health of Cambodian preschool children is poor [10, 11, 13, 38]. In this study, the majority of the children (61.5%) had ECC by the time they reached the age of three years. All caries was untreated, and ECC was a predictor of underweight. Untreated ECC can have impacts on the children and their families [38]. It can result in pain and infection, reduce dietary intake, and cause financial hardship to the family if treatment is sought. A previous study has shown that providing dental treatment for ECC had a significant effect on weight catch-up [42]. However, in Cambodia, few preschool children access dental care, some of which in other countries would be provided under general anesthesia. In addition, dental clinics and oral health personnel are scarce in rural Cambodia. In the present study population, only 25% of caregivers had started brushing after the eruption of the first teeth, and >50% of children consumed sugary foods and drinks several times a week. This indicates that ECC prevention, including tooth brushing instruction, dietary advice and fluoride varnish applications from an early age should be prioritized. A recent community-based intervention which included oral health education and fluoride varnish by non-dental health workers in rural Cambodia led to a lower ECC prevalence in children aged two years [43]. Regarding nutritional interventions, adaptation of age-appropriating feeding practices from an early age has contributed to improving child growth and development in Cambodia [44]. This integrated approach could help to reduce the incidence of both ECC and underweight among young rural children.

This study has several limitations. First of all, only children who attended for health check-ups were included in the study. These caregivers may have had a higher level of health concern for their child compared to other mothers, resulting in selection bias. Second, the study was conducted on a small population size in one rural part of Cambodia, and since it was cross-sectional, causal relationships between ECC and underweight could not be established. Further longitudinal studies should be conducted to investigate the role of ECC in underweight.

## Conclusions

In conclusion, the prevalence of underweight and ECC were high in this group of one to three years old children, and both increased with age. The findings of this study suggest that low birthweight, malnutrition based on MUAC scores and ECC were predictors of underweight in this population. Comprehensive health and nutrition programs targeting children during the weaning period should be conducted in order to address underweight and ECC in Cambodia.
